# Loss of *Drosophila* ribosomal protein S6 kinase II causes mitochondrial dysfunction and cell death

**DOI:** 10.1242/dmm.052374

**Published:** 2025-08-19

**Authors:** Ting Deng, Lajos Kalmar, Samantha Loh, Olivier E. Pardo, L. Miguel Martins

**Affiliations:** ^1^ MRC Toxicology Unit, University of Cambridge, Gleeson Building, Tennis Court Road, Cambridge CB2 1QR, UK; ^2^ Department of Pathology, University of Cambridge, Tennis Court Road, Cambridge CB2 1QP, UK; ^3^ Division of Cancer, Imperial College London, Hammersmith Hospital Campus, Du Cane Road, London W12 ONN, UK

**Keywords:** Mitochondria, *Drosophila*, Cell death, Kinase

## Abstract

Mitochondria are dynamic organelles that are critical for energy production in high-demand tissues, such as the brain and muscle, with fusion and fission maintaining network integrity. The dysregulation of these processes underlies pathologies, such as neurodegenerative diseases. Ribosomal S6 kinases (RSK1–4) are effectors of extracellular signal-regulated kinases (ERKs), with roles in cell survival and metabolism. Here, we show that RSKs are essential for mitochondrial health. In human cells, siRNAs targeting any RSK isoform (RSK1–4) induced mitochondrial fragmentation and reduced viability. In *Drosophila melanogaster*, CRISPR-mediated loss of S6kII (the sole RSK orthologue) caused mitochondrial dysfunction and tissue degeneration in high-energy-demand organs, including the indirect flight muscle and brain, accompanied by autophagic activation. Notably, we rescued these defects by expressing human RSK4, underscoring functional conservation. Our findings establish RSKs as critical regulators of mitochondrial integrity, linking ERK signalling to organelle dynamics. This work identifies RSKs as regulators of mitochondrial health in energy-demanding tissues, providing insights into the mechanisms underlying neurodegeneration and strategies to target ERK/RSK-driven mitochondrial dysfunction.

## Teaser

**Summary:** We show in *Drosophila* and in human cells that loss of ribosomal S6 kinases causes mitochondrial dysfunction and cell death, providing insights into the mechanisms underlying neurodegeneration.

## INTRODUCTION

Mitochondria supply most of the cellular energy and are essential in high-energy-demanding animal tissues, such as the brain and muscle (reviewed in Celardo et al., 2014; Zong et al., 2024). These organelles produce most of the energy required for cellular function, such as adenosine triphosphate (ATP). Mitochondria are dynamic organelles that merge and divide by fusion and fission, respectively. These processes are essential for maintaining a healthy mitochondrial network that supports cell viability. Mitochondrial fusion enables the transfer of metabolites, proteins and mitochondrial DNA (mtDNA) between two mitochondria to ensure mitochondrial health. Mitochondrial fission supports the even distribution of mitochondria during cell division and helps isolate and remove damaged mitochondria (reviewed in de Castro et al., 2011). Defects in mitochondrial function and dynamics are hallmarks of diseases, such as neurodegenerative disorders and cancers (reviewed in Chen et al., 2023). Mitochondrial dysfunction is characterised by impaired oxidative phosphorylation, the excessive generation of reactive oxygen species (ROS) and morphological alterations. These changes contribute to the pathogenesis of disorders affecting high-energy-demanding tissues, such as the brain and muscles (reviewed in Celardo et al., 2014).

Kinase signalling pathways, including the mitogen-activated protein kinase (MAPK) signalling cascade, play key roles in regulating mitochondrial function and dynamics and cell survival (reviewed in Cook et al., 2017). Three MAPK pathways with different cellular functions are well described: the ERK pathway, the c-Jun N-terminal kinase (JNK) pathway and the p38 MAPK pathway. The ERK pathway plays important roles in regulating cell proliferation, differentiation and survival. This pathway regulates mitochondrial function by controlling mitochondrial dynamics and biogenesis, metabolic reprogramming and the oxidative stress response. In particular, ERK1 (also known as MAPK3) and ERK2 (also known as MAPK1) can alter mitochondrial dynamics by directly phosphorylating mitofusin 1 (MFN1) at Thr562 (Pyakurel et al., 2015) and dynamin-related protein 1 (Drp1; also known as DENR in human) at Ser616 (Kashatus et al., 2015). In neurodegenerative diseases, aberrant ERK activity has been linked to mitochondrial fragmentation, oxidative stress and neuronal death. For example, in Alzheimer's disease models, ERK–Drp1 signalling is proposed to contribute to cytotoxicity (Yuan et al., 2021).

Despite extensive studies on ERK and its role in neurodegeneration, the downstream effectors that mediate its impact on mitochondrial function remain unknown. Ribosomal S6 kinases (RSKs) constitute a family of serine/threonine kinases that act as negative regulators of ERK signalling.

RSKs comprise four family members: RSK1 (also known as RPS6KA1), RSK2 (also known as RPS6KA3), RSK3 (also known as RPS6KA2) and RSK4 (also known as RPS6KA6). They play crucial roles in processes, such as cell proliferation, survival and metabolism (reviewed in Lara et al., 2013), contain two distinct kinase domains and act as effectors of ERK signalling (reviewed in Cronin et al., 2021). Whereas RSK1, RSK2 and RSK3 are activated in cells upon growth factor stimulation, RSK4 is constitutively active even in serum-starved cells (Dummler et al., 2005).

*Drosophila* contains a single RSK gene in its genome, *S6kII* (Wassarman et al., 1994). In *Drosophila*, S6kII inhibits ERK signalling by acting as a cytoplasmic anchor for ERK (Kim et al., 2006).

Mitochondrial dynamics and function, which are regulated by ERK signalling, control cell fate decisions, such as apoptosis (reviewed in Cook et al., 2017), and RSKs modulate ERK signalling pathways (reviewed in Cook et al., 2017). We therefore investigated the consequences of the removal of these kinases for mitochondrial health. We show that the downregulation of RSKs in cultured human cells causes mitochondrial fragmentation and a decrease in cell viability. In addition, we observed that the removal of this kinase in adult *Drosophila* leads to mitochondrial dysfunction and tissue deterioration within high-energy-demanding organs, such as the muscles and brain. This deficiency also triggered autophagy, possibly to offset mitochondrial damage. Finally, constitutively expression of RSK4 in flies rescued the mitochondrial defects observed upon loss of S6kII. Our findings show that RSKs play a key role in mitochondrial health.

## RESULTS

### Suppression of RSKs in mammalian cells results in mitochondrial fragmentation and lower cell viability

**Fig. 1.**
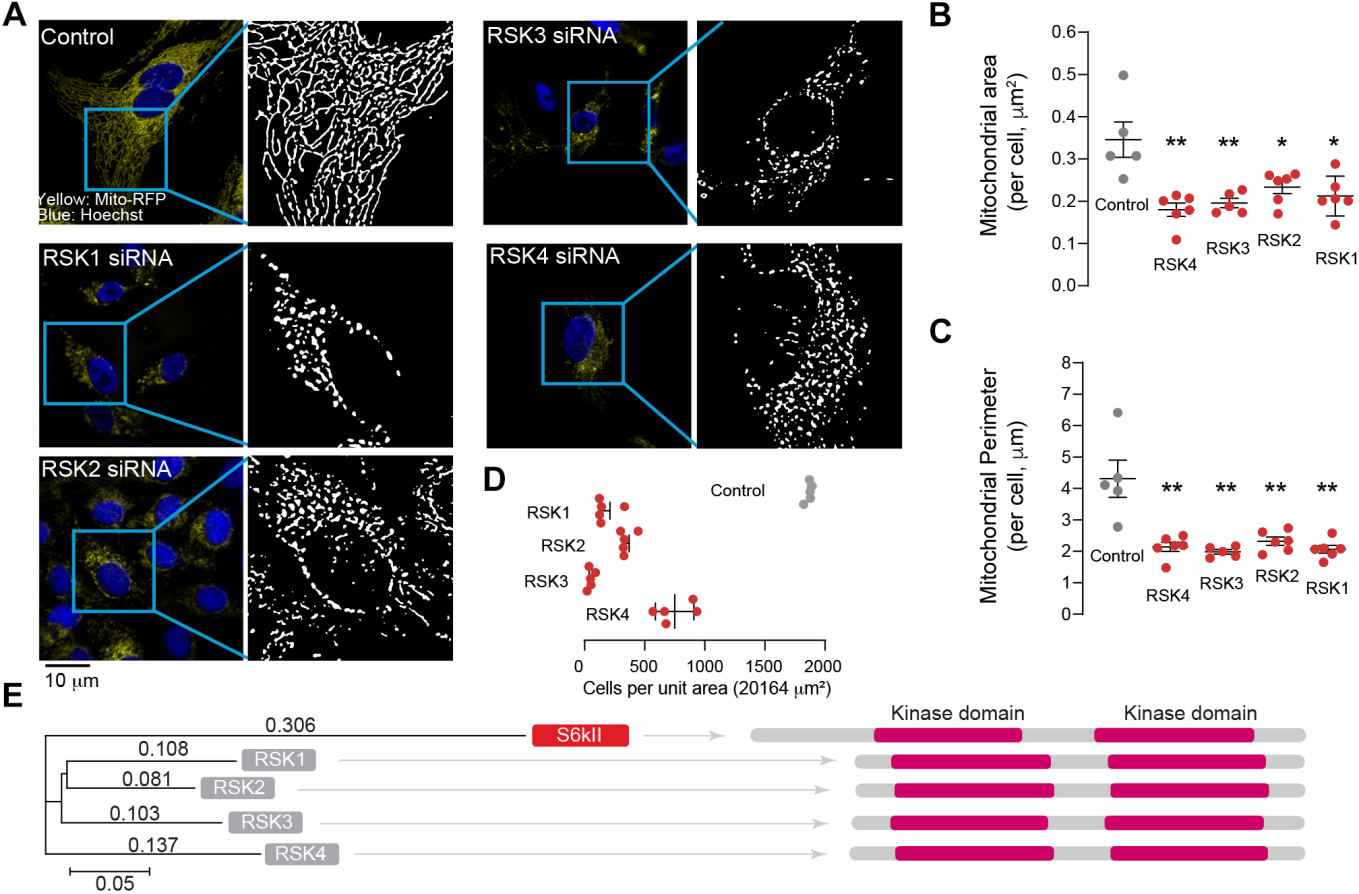
**Suppression of RSK1–4 causes mitochondrial defects in cultured cells.** (A–C) RNA interference (RNAi)-mediated suppression of RSKs causes mitochondrial fragmentation in U2OS cells. (A) Representative images (left panels) of cells stably expressing mitochondrion-targeted RFP and transfected with different siRNAs (see Materials and Methods). The right panels show examples of the mitochondrial network thresholded on a binary scale and subjected to an automated analysis. (B,C) Measured mitochondrial area (B) and perimeter (C) (means±s.e.m.; *P≤0.05, **P≤0.01, one-way ANOVA followed by Dunnett's post hoc test). Each dot corresponds to an individual cell analysed. (D) Suppressing the expression of mammalian RSKs in cultured cells decreases cell viability (means±s.e.m.; one-way ANOVA followed by Dunnett's post hoc test). (E) Comparative analysis between *Drosophila* S6kII and human RSKs (RSK1, RSK2, RSK3 and RSK4). The phylogenetic analysis (left panel) was performed using the neighbour joining method. The numbers correspond to the distance between proteins in phylogenetic units, where a value of 0.05 corresponds to a difference of 5% between two sequences. Alignment of the structural domains of *Drosophila* S6kII and human RSKs (right panel) was performed using MOTIF. The N-terminal (left) and C-terminal (right) kinase domains, mapped by MOTIF, are highlighted in pink.

RSKs regulate multiple intracellular signalling pathways, including the ERK1/2 MAPK signalling pathway (Romeo et al., 2012). This pathway, in turn, regulates cell fate decisions, particularly the BCL2-regulated cell-intrinsic mitochondrial pathway of apoptosis (Cook et al., 2017). Because the mitochondrial pathway of apoptosis is also associated with the fragmentation of the mitochondrial network (Cook et al., 2017), we analysed mitochondrial morphology in cultured human cells upon RNA interference (RNAi)-mediated suppression of different human RSKs. We found that suppressing any of these four kinases caused the fragmentation of the mitochondrial network (Fig. 1A–C) and a decrease in cell number (Fig. 1D). We conclude that suppressing RSKs leads to mitochondrial fragmentation and cell death or defects in cell proliferation.

### Loss of *S6kII* causes mitochondrial dysfunction

The four different RSKs in humans (*RSK1*, *RSK2*, *RSK3* and *RSK4*) have a single orthologue in fruit flies (*Drosophila*), *S6kII* (Wassarman et al., 1994). They all share common features, such as two separate kinase domains separated by a linker region (Fig. 1E).

**Fig. 2.**
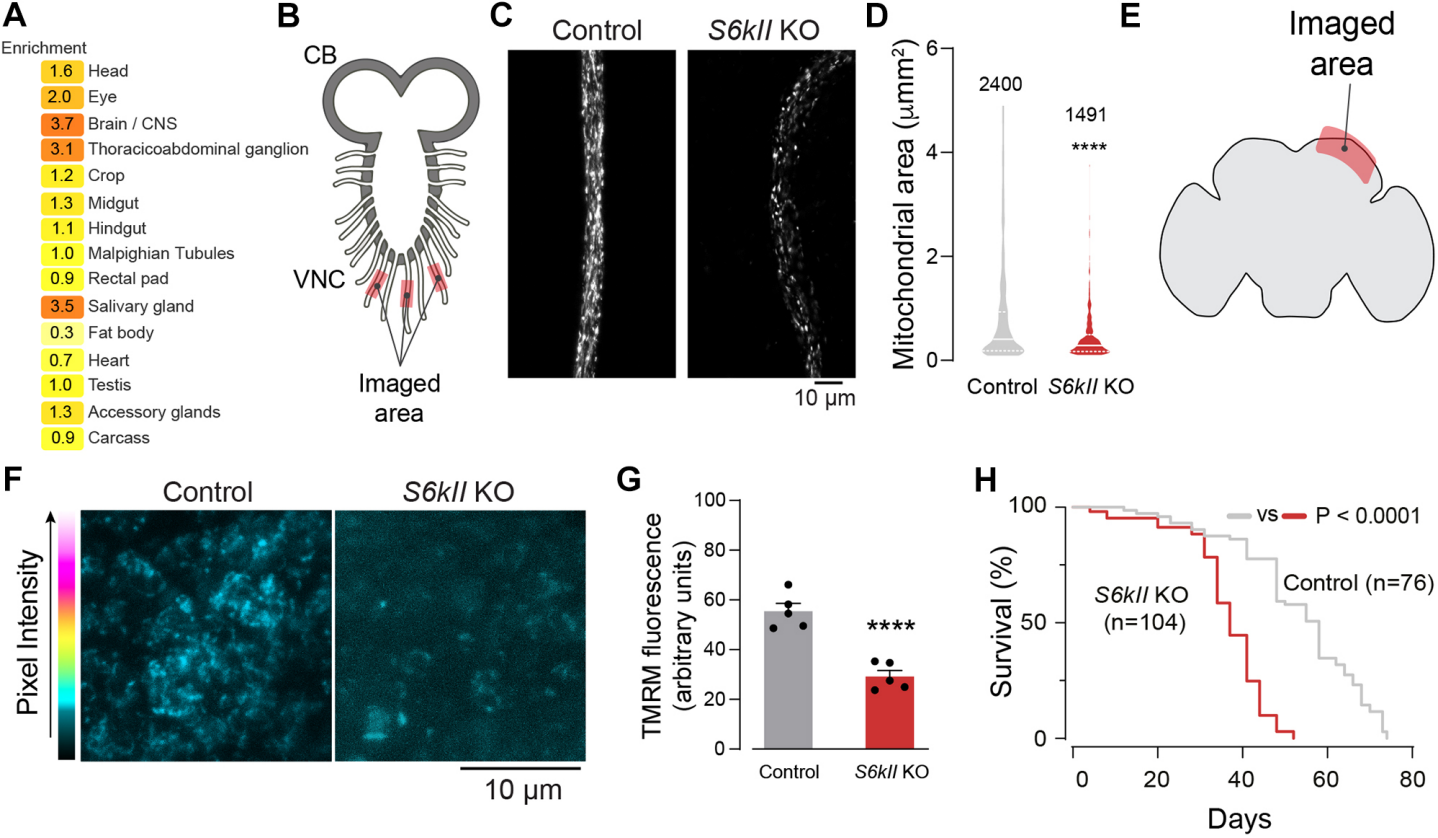
***Drosophila S6kII* knockout (KO) flies show mitochondrial fragmentation and decreased mitochondrial function in neuronal tissues.** (A) Expression levels of *S6kII* in different tissues of adult *Drosophila* male flies. Enrichment is defined as a measure of the abundance of *S6kII* in a particular tissue relative to the whole fly. Results from a query to FlyAtlas 2 (Krause et al., 2022). (B) Schematic diagram of the *Drosophila* larval central nervous system (CB, central brain; VNC, ventral nerve cord). (C,D) The loss of S6kII results in mitochondrial fragmentation. (C) Confocal analysis of mitoGFP fluorescence in larval VNC axons. (D) Quantification of mitochondrial length is shown as a combined violin and box plot (medians with interquartile ranges; ****P≤0.0001, two-tailed unpaired *t*-test). (E) A schematic of the adult *Drosophila* brain in the sagittal orientation. (F,G) The loss of mitochondrial membrane potential (Δψm) in the brain mitochondria of S6kII KO flies. (F) Representative confocal images of whole-mounted brains showing neurons loaded with tetramethylrhodamine methyl ester (TMRM). The intensity levels of TMRM are mapped using the Fire Lookup Table in Fiji. (G) Quantification of the loss of Δψm (means±s.e.m.; ****P≤0.0001, two-tailed unpaired *t*-test). (H) Decreased lifespan in S6kII KO flies. Fly viability was scored over a period of 80 days. Genotypes: *w*; *elavGal4/+; UASmitoGFP/+* (control), *S6kII^KO^; elavGal4/+; UASmitoGFP/+* (S6kII KO) (C,D); *w^1118^; +; +* (control), *S6kII^KO^; +; +* (S6kII KO) (F–H).

*Drosophila* is a useful organism to model mitochondrial function and cell death pathways. An analysis of the expression levels of *S6kII* in adult flies showed increased enrichment of this transcript in neuronal tissues, such as the central nervous system and the thoracicoabdominal ganglion (Fig. 2A), mirroring the reported expression of its human orthologues (reviewed in Ziyi et al., 2024). We used a null mutant allele of *S6kII* generated using CRISPR technology [S6kII knockout (KO) flies] to study the potential role of RSKs in regulating mitochondrial function (Mast and DiPrimio, 2019). Because the suppression of RSKs in mammalian cells altered mitochondrial morphology, we analysed mitochondria from the larval ventral nerve cord of S6kII KO flies (Fig. 2B–D) and observed that they were fragmented. Next, we examined the functional state of mitochondria in adult S6kII KO flies. The health and function of mitochondria can be assessed by measuring the mitochondrial membrane potential (Δψm). We measured the Δψm in the brains of adult S6kII KO flies using tetramethylrhodamine methyl ester (TMRM) and detected a decrease in the Δψm (Fig. 2E–G). We next studied the flies’ lifespan and found that S6kII KO flies had a reduced lifespan (Fig. 2H). We conclude that the loss of S6kII in flies causes mitochondrial dysfunction and reduced lifespan.

### Absence of S6kII in flies results in increased ROS levels in the brain and age-related decreases in ATP levels and antioxidant defences

**Fig. 3.**
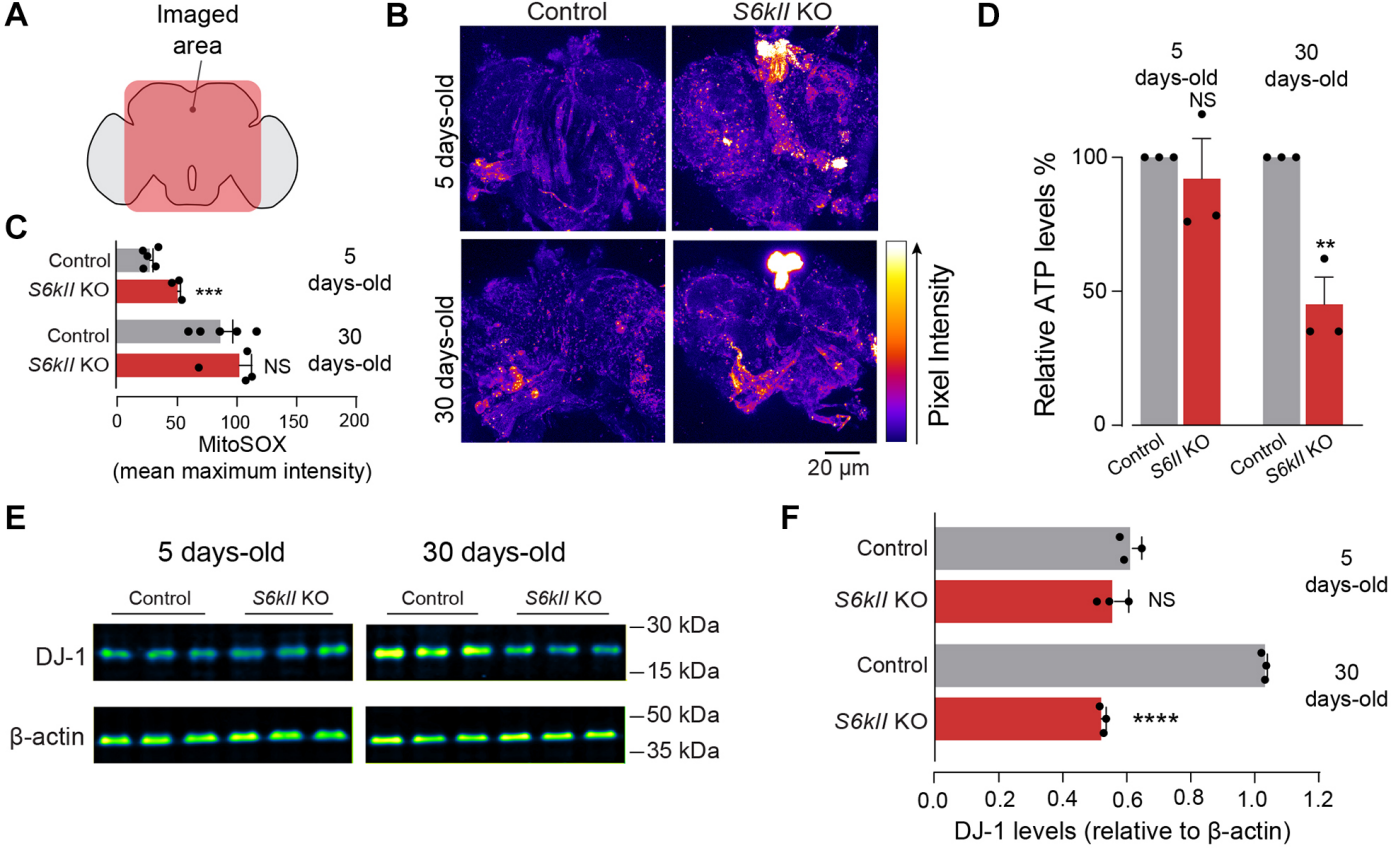
***S6kII* KO flies show increased levels of reactive oxygen species (ROS) and decreased ATP levels in the brain.** (A) A schematic of the adult *Drosophila* brain in sagittal orientation, indicating the approximate analysed region. (B,C) Increased levels of mitochondrial ROS were detected in the brains of S6kII KO flies. Representative images (B) with MitoSOX Red intensity levels mapped using the Fire Lookup Table in Fiji and quantification (C) [means±s.e.m.; NS, not significant (*P*>0.05); ***P≤0.001, two-tailed unpaired *t*-test]. (D) Lower levels of ATP detected in the brains of 30-day-old S6kII KO flies [means±s.e.m.; NS, not significant (*P*>0.05); **P≤0.01, two-tailed unpaired *t*-test]. (E,F) Lower DJ-1 protein levels were detected in brains of *S6kII* KO flies. Representative cropped immunoblots (E) with false colour rendering by Fiji and corresponding quantification (F), with the levels of DJ-1 normalised to those of β-actin [means±s.e.m.; NS, not significant (*P*>0.05); ****P≤0.001, two-tailed unpaired *t*-test]. Genotypes: *w^1118^; +; +* (control), *S6kII^KO^; +; +* (*S6kII* KO) (B–F).

Impaired mitochondrial function is associated with the production of toxic ROS, which can activate cell death pathways. We measured mitochondrial ROS levels in the brains of S6kII KO flies using MitoSOX Red, a mitochondrial superoxide indicator, and detected an increase in mitochondrial ROS levels in young (5-day-old) flies (Fig. 3A–C). As mitochondria are responsible for the generation of most of the cellular energy available as ATP, we next measured ATP levels in the brains of S6kII KO flies. We found that the loss of S6kII decreased the ATP levels in aged (30-day-old) flies (Fig. 3D). DJ-1 (also known as PARK7) protein acts as a sensor for oxidative stress and antioxidant activity, and mutations in this protein cause an inherited form of Parkinson's disease (Dolgacheva et al., 2019). *Drosophila* has two homologues of human DJ-1: Dj-1alpha and Dj-1beta. Dj-1alpha is expressed predominantly in the testis, whereas Dj-1beta is present in most tissues (Menzies et al., 2005). We probed brain protein lysates from S6kII KO flies with an antibody against DJ-1 and detected a decrease in the level of immunoreactive protein in ageflies (Fig. 3E,F).

We conclude that the loss of *S6kII* results in increased mitochondrial ROS levels in the brains of young flies; in older flies, this effect is linked to diminished energy levels and antioxidant defences.

### Loss of *S6kII* causes apoptosis in the brain and muscles of adult flies

**Fig. 4.**
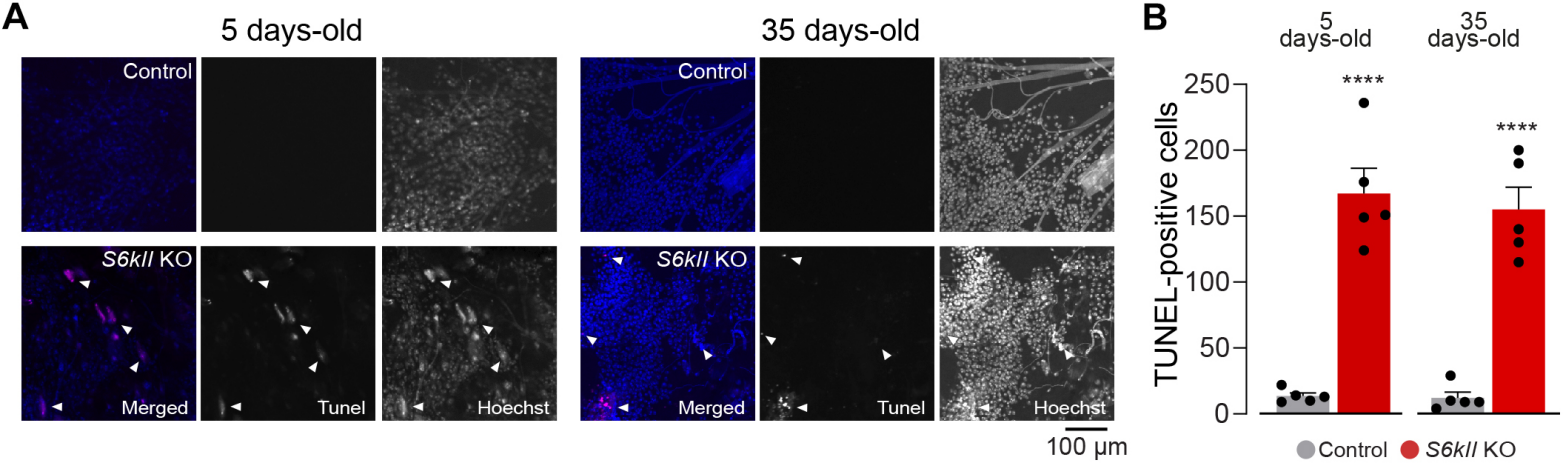
**Loss of *Drosophila S6kII* causes cell death in the adult *Drosophila* brain.** (A) Representative images of adult fly brains stained with Hoechst and assayed for apoptosis using the terminal deoxynucleotidyl transferase dUTP nick end labelling (TUNEL) assay. The merged panels show DNA fluorescence (Hoechst) in blue and TUNEL-positive cells in red. The individual channels are also shown to the right of the merged channel. Arrowheads point to examples of TUNEL-positive cells. (B) Quantification of the TUNEL-positive cells revealed an increase in apoptosis in the brains of both young and aged S6KII KO flies (means±s.e.m.; ****P≤0.001, two-tailed unpaired *t*-test). Dots correspond to individual brains. Genotypes: *w^1118^; +; +* (control), *S6kII^KO^; +; +* (S6kII KO) (A,B).

**Fig. 5.**
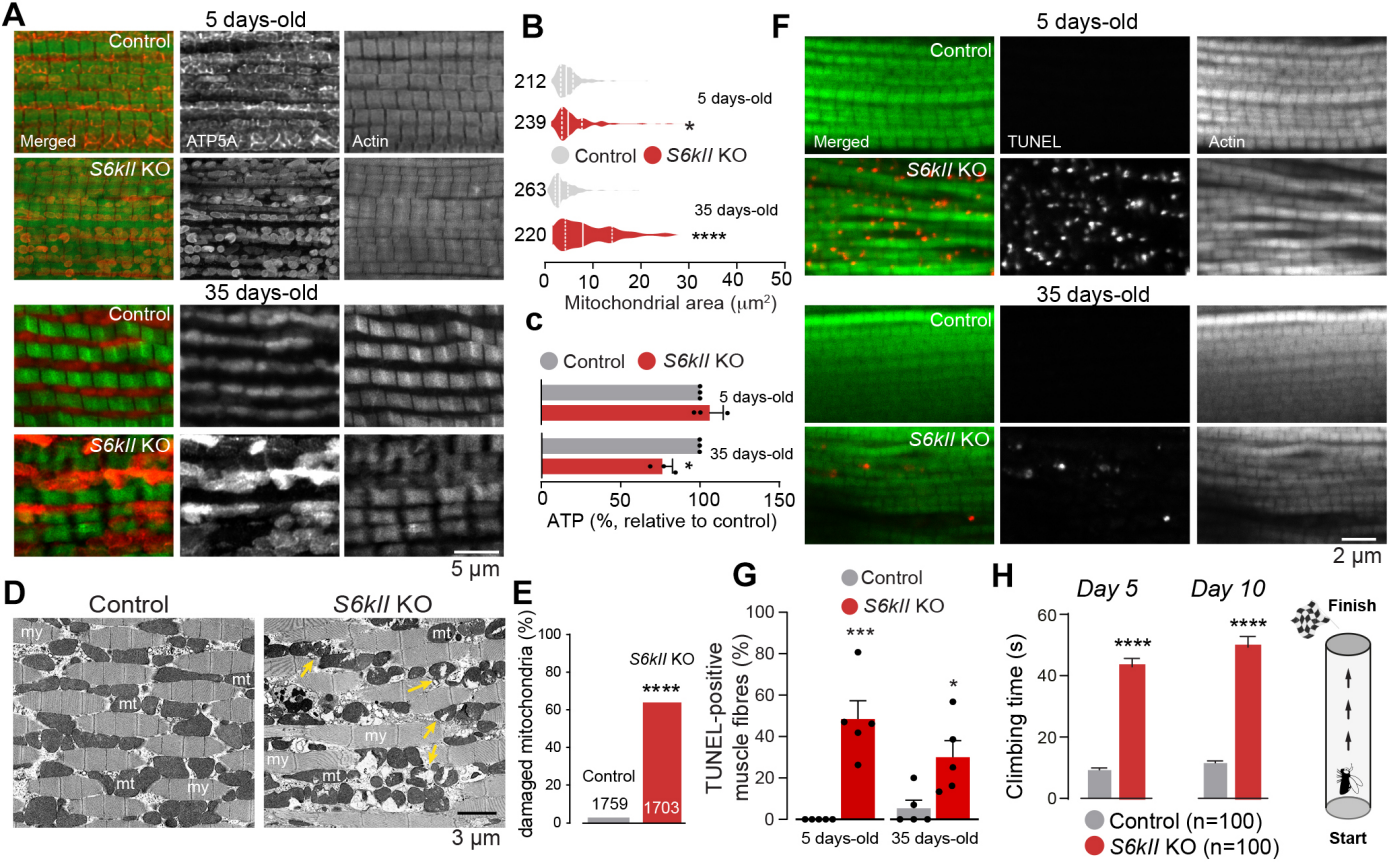
**Loss of *S6KII* causes muscle defects and motor impairment.** (A,B) Changes in mitochondrial morphology in the indirect flight muscle of *S6kII* KO flies. (A) Representative confocal images of the indirect flight muscle of flies stained with an antibody against mitochondrial ATP5A and fluorescent phalloidin to stain actin fibres. (B) Quantitative analysis revealed an increase in the mitochondrial area in S6kII KO flies (means±s.e.m.; *P≤0.05, ****P≤0.0001, two-tailed unpaired *t*-test). (C) Lower ATP levels were detected in the indirect flight muscle of S6kII KO flies (means±s.e.m.; *P≤0.05, two-tailed unpaired *t*-test). (D,E) Ultrastructural defects in the indirect flight muscles of S6kII mutant flies. The tissues were analysed using transmission electron microscopy. Yellow arrows indicate defective mitochondria (D), which are quantified (E); ****P≤0.0001, two-sided chi-square test. mt, mitochondria; my, myofibrils. (F,G) Increased cell death in the indirect flight muscles of S6kII KO flies. (F) Representative confocal images of muscles stained with TUNEL for apoptosis. (G) Quantification of TUNEL-positive nuclei (means±s.e.m.; ***P≤0.001, two-tailed unpaired *t*-test). (H) Motor impairment in S6kII KO flies. Climbing performance of adult control and S6kII KO flies at the indicated ages. The total number of flies tested per group is indicated on the graph (means±s.e.m.; ****P≤0.0001, two-tailed unpaired *t*-test). Genotypes: *w^1118^; +; +* (control), *S6kII^KO^; +; +* (S6kII KO) (A–H).

Elevated ROS levels within cells damage proteins, nucleic acids, lipids, membranes and organelles, resulting in apoptotic cell death. The terminal deoxynucleotidyl transferase dUTP nick end labelling (TUNEL) assay is a method for detecting DNA fragmentation by labelling the 3′-hydroxyl termini in the double-strand DNA breaks generated during apoptosis. We therefore used this assay to measure the levels of apoptosis in the brains of flies lacking *S6kII* and observed an increase in the number of apoptotic cells in S6kII KO flies (Fig. 4A,B). Mitochondrial defects lead to the degeneration of tissues with high energy requirements in flies, such as the indirect flight muscle (IFM) (Pimenta de Castro et al., 2012). An analysis of the IFM of S6kII KO flies revealed that the loss of this kinase resulted in morphological defects in the mitochondria (Fig. 5A.B), an age-dependent decrease in ATP levels (Fig. 5C) and increased mitochondrial damage (Fig. 5D,E). This mitochondrial damage was associated with an increase in apoptosis (Fig. 5F,G). Because mitochondrial defects in *Drosophila* muscles cause their degeneration, leading to motor defects, we next assayed the motor performance of S6kII KO flies using a climbing assay. We found that the loss of *S6kII* resulted in decreased climbing performance (Fig. 5H).

We conclude that the loss of *S6kII* impairs mitochondrial health in muscles, causing cell death and motor dysfunction.

### Global proteomic changes in S6kII KO flies

**Fig. 6.**
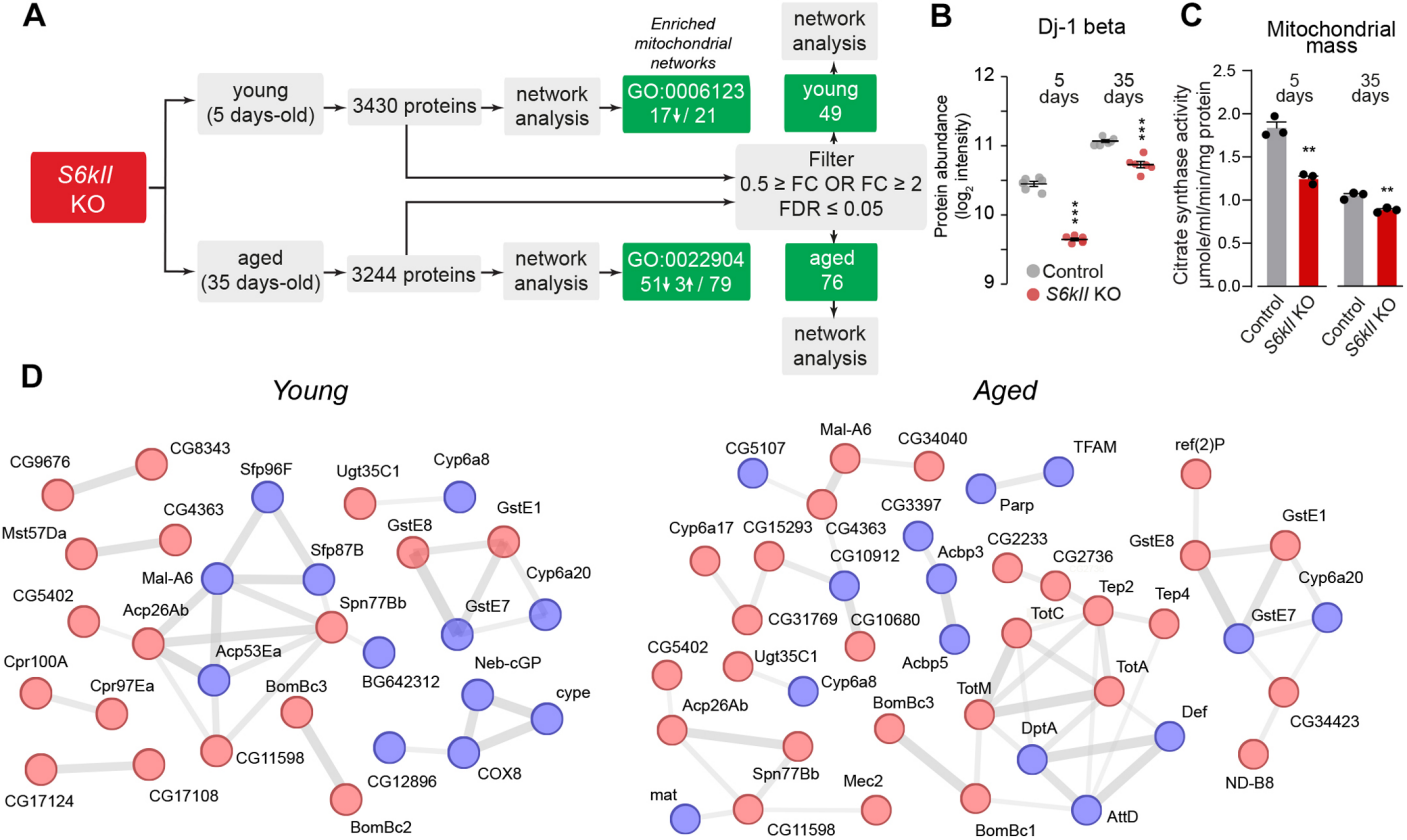
**Proteomic analysis of *S6kII* KO flies shows a decrease in the expression of mitochondrial proteins.** (A) Workflow used to characterise the proteins that were altered in S6kII KO flies. Proteins were filtered by the adjusted false discovery rate (FDR) and fold change (FC) values of 0.05 and ±2-fold, respectively, to identify differentially expressed targets. (B) Decreased levels of Dj-1beta (UniProt accession code Q9VA37) were detected in S6kII KO flies. Protein levels were measured by mass spectrometry (means±s.e.m.; ***P≤0.001, two-tailed Student's *t*-test). This figure panel is related to Table S1. (C) The loss of S6kII is associated with a decrease in the mitochondrial mass, as assessed by measuring the activity of the mitochondrial matrix enzyme citrate synthase in adults (means±s.d.; **P≤0.01, two-tailed unpaired *t*-test, compared with the control). (D) STRING network analysis of age-dependent proteomic alterations in S6kII KO flies. Red and blue nodes represent upregulated and downregulated proteins, respectively. Clusters were obtained via STRING with a medium confidence score (0.400), and the line thickness of the edges corresponds to the strength of the data supporting the connection between joined nodes. Genotypes: *w^1118^; +; +* (control), *S6kII^KO^; +; +* (S6kII KO) (A–D).

RSKs can influence the activity of the mammalian target of rapamycin (mTOR) pathway. This pathway regulates eukaryotic cell growth and controls various processes, including protein synthesis, mitochondrial biogenesis and autophagy (reviewed in Liu and Sabatini, 2020). The target of rapamycin pathway is present in *Drosophila*, and this insect contains a unique TOR (dTOR)-encoding gene (reviewed in Frappaolo and Giansanti, 2023). Because S6kII is an orthologue of RSKs, and because of the roles of these kinases in the regulation of protein translation, autophagy and mitochondrial biogenesis, we next analysed global alterations in the proteome of S6kII KO flies (Fig. 6A). We measured the levels of more than 3000 proteins in both young and aged flies (Fig. 6A; Tables S1 and S2). A Gene Ontology (GO) analysis of the global proteomic changes revealed the downregulation of most proteins mapped to mitochondrial GO identifiers (GO:0006123, mitochondrial electron transport; GO:0022904, respiratory electron transport chain) (Fig. 6A; Tables S3 and S4). This unbiased proteomic analysis also revealed a decrease in Dj-1beta expression (Fig. 6B), confirming the results from the western blot analysis of S6kII KO flies (Fig. 3E,F).

We measured the activity of the mitochondrial matrix enzyme citrate synthase, an indirect indicator of mitochondrial density, to investigate the effect of *S6kII* loss on mitochondrial density in flies (Magwere et al., 2006). Citrate synthase activity in the S6kII KO flies was lower than that in the control flies (Fig. 6C). The global reduction in the levels of mitochondrial proteins, as shown by our proteomic analysis and this observation, indicated that the loss of S6kII decreased the mitochondrial mass. Next, we focused our proteomic analysis on the most significantly altered proteins in both young and aged S6kII KO flies and performed a network analysis of the altered proteome (Fig. 6A). This analysis confirmed alterations in members of the protein networks involved in antioxidant defences (GstE1, GstE7 and GstE8), mitophagy [Ref(2)P (de Castro et al., 2013)] and mitochondrial biogenesis (TFAM). We also observed alterations in network components related to innate immunity (TotA and TotC) that were previously reported by our group to be associated with mitochondrial damage (Fedele et al., 2022) (Fig. 6D; Tables S5 and S6).

We conclude that the loss of *S6kII* in adult flies causes alterations in protein networks involved in mitochondrial quality control mechanisms.

### S6kII KO flies have increased levels of components of the autophagy machinery

Mitochondria are dynamic organelles that are constantly reshaped through fusion and fission. Cells use mitochondrial fission to target damaged mitochondria for degradation via autophagy. This degradation involves the PINK1-dependent recruitment of the ubiquitin ligase parkin to defective mitochondria. Parkin then ubiquitinates mitochondrial proteins that work as adaptors for p62/SQSTM1, the human orthologue of Ref(2)P. Complexes between ubiquitin and these adaptors promote the translocation of defective mitochondria to the autophagosome for removal (reviewed in de Castro et al., 2011).

**Fig. 7.**
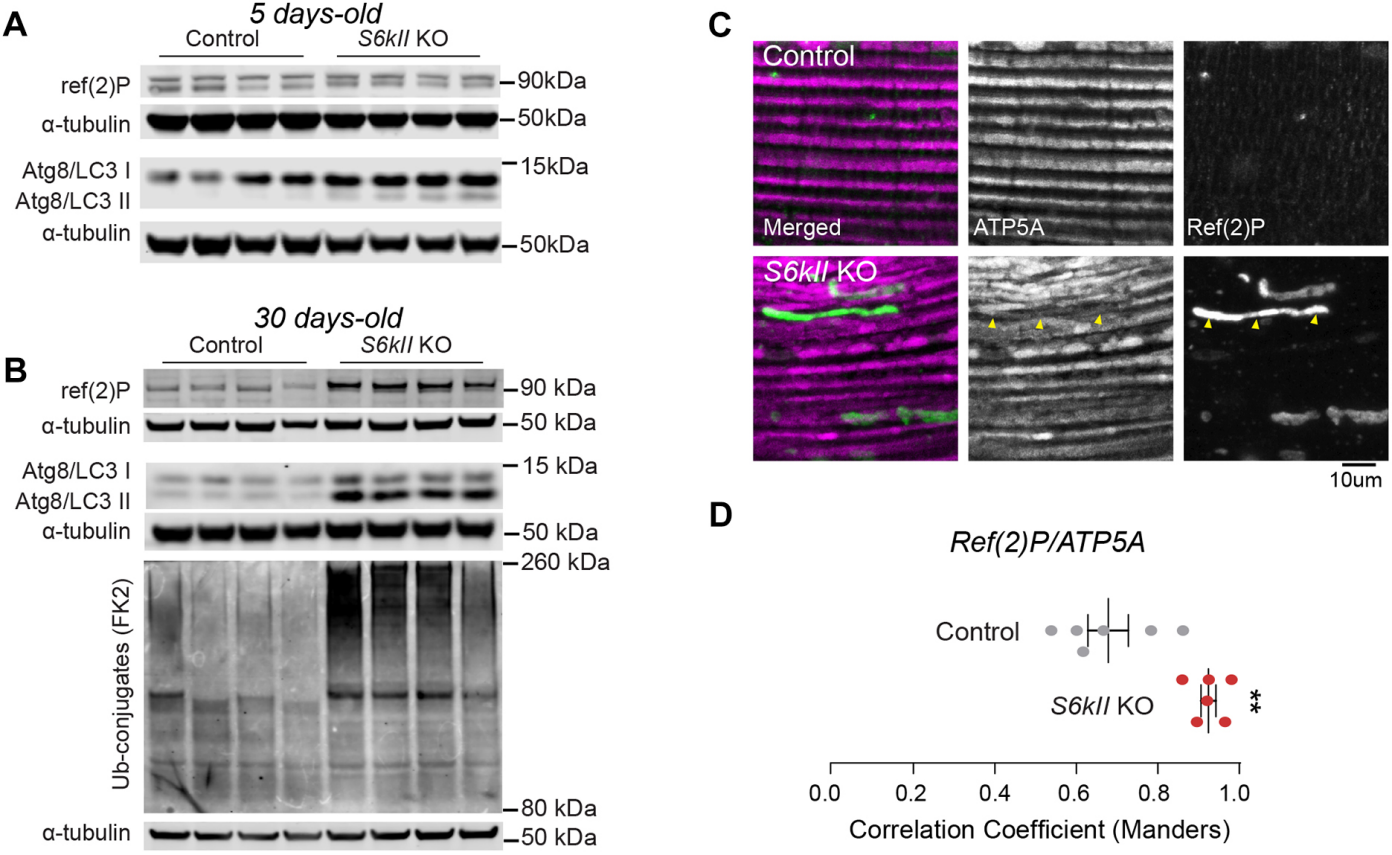
***S6kII* KO flies show an increase in the expression of autophagy markers.** (A,B) Increased levels of Ref(2)P, ATG8 and protein ubiquitination in 5-day-old (A) and 30-day-old (B) S6kII KO flies. Representative cropped immunoblots probed with the indicated antibodies. Atg8/LC3 I and Atg8/LC3 II correspond to the migration patterns of the nonlipidated and lipidated forms of the protein, respectively. (C,D) Colocalisation of Ref(2)P and mitochondria in the muscle tissue of aged S6kII KO flies. (C) Representative confocal images of the indirect flight muscle of flies stained with antibodies against mitochondrial ATP5A and Ref(2)P, with yellow arrowheads indicating an example of a mitochondrion that is positive for Ref(2)P. (D) The colocalisation of Ref(2)P-positive clusters with mitochondria was analysed using Manders correlation analysis (Manders et al., 1993) with the Coloc 2 plug-in of Fiji (means±s.e.m.; **P≤0.01, two-tailed unpaired *t*-test). Genotypes: *w^1118^; +; +* (control), *S6kII^KO^; +; +* (S6kII KO) (A–D).

Because we detected increased levels of Ref(2)P in S6kII KO flies and a generalised loss of mitochondrial proteins, we next assessed whether the loss of *S6kII* was linked to increased autophagy. Atg8a is the main *Drosophila* orthologue of mammalian microtubule-associated protein 1A/1B-light chain 3 (LC3; also known as MAP1LC3) family proteins (Szabo et al., 2023). LC3 is a soluble cytosolic protein. Autophagy involves the sequestration of cytosolic proteins and organelles by autophagosomes. This process involves the conversion of cytosolic LC3 (LC3-I) to the phosphatidylethanolamine-conjugated LC3-II form. Subsequently, LC3-II translocates to autophagosomal membranes, where it engages in autophagic processes (reviewed in Tanida et al., 2008). We probed protein lysates from S6kII KO flies with an antibody that recognises Atg8a/LC3 and detected increased levels of both LC3-I and LC3-II in young and aged flies. We also confirmed the upregulation of the levels of Ref(2)P in aged flies, together with an increase in the levels of proteins that are immunoreactive with an anti-ubiquitin antibody (Fig. 7A,B). Because Ref(2)P participates in mitophagy, the autophagy-mediated elimination of damaged mitochondria (de Castro et al., 2013), we next investigated its distribution within the IFM of S6kII KO flies. We found that Ref(2)P colocalised with a subset of mitochondria in the IFM that showed decreased immunoreactivity for ATP5A (also known as Blw), a mitochondrial protein (yellow arrowheads in Fig. 7C,D).

We conclude that the loss of *S6kII* in adult flies promotes the upregulation of autophagic pathways that act to degrade defective mitochondria.

### Expression of S6kII or RSK4 rescues mitochondrial defects in S6kII KO flies

**Fig. 8.**
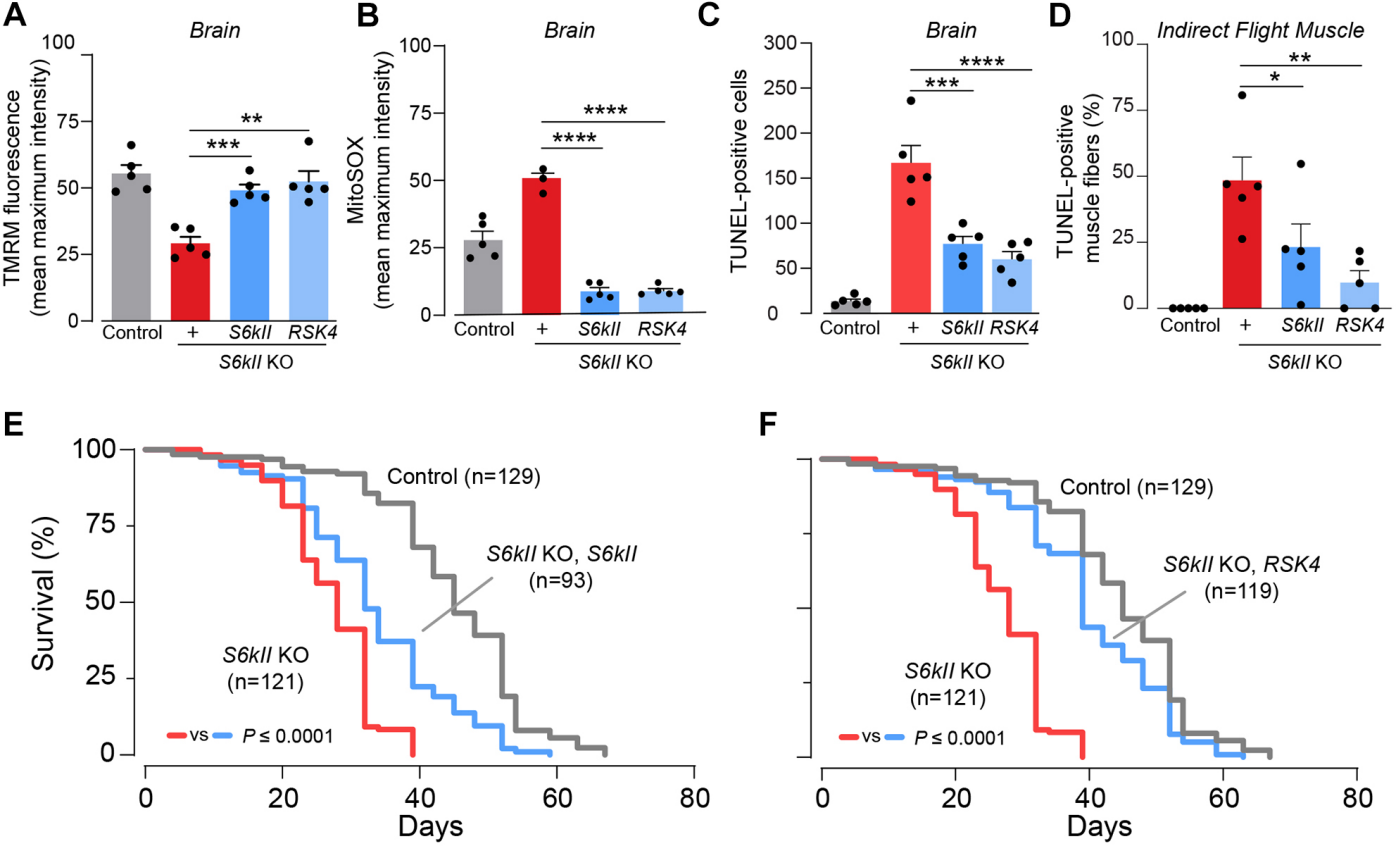
**Overexpression of *S6kII* or *RSK4* rescues the mitochondrial dysfunction in *S6kII* KO flies.** (A–D) The expression of either *Drosophila S6kII* or human *RSK4* in S6kII KO flies increases the Δψm (A; means±s.e.m.; **P≤0.01, ***P≤0.001, one-way ANOVA followed by Dunnett's post hoc test), decreases mitochondrial ROS levels (B; means±s.e.m.; ****P≤0.0001, one-way ANOVA followed by Dunnett's post hoc test) and suppresses cell death (C,D; means±s.e.m.; *P≤0.05, **P≤0.01, ***P≤0.001, ****P≤0.0001, one-way ANOVA followed by Dunnett's post hoc test) in 5-day-old adult male flies. (E,F) The lifespan of S6kII KO flies increased upon expression of *S6kII* (E) or *RSK4* (F). Genotypes: *w^1118^; +; +* (control, grey bar), *S6kII^KO^; elavGal4+; +* (red bar), *S6kII^KO^; elavGal4/+; UAS S6kII/+* (dark-blue bar), *S6kII^KO^; elavGal4/+; UAS RSK4/+* (light-blue bar) (A–F).

Next, we determined the effect of the increased ubiquitous expression of S6kII or one of its human orthologues in S6kII KO flies. Four human orthologues of S6kII have been identified (Fig. 1E), and, among these four, RSK4 is not subjected to activation by growth factor stimulation (Dummler et al., 2005). We therefore created an engineered version that was codon optimised (see Materials and Methods) for constitutive expression in *Drosophila*. We found that expressing either *S6kII* or *RSK4* in S6kII KO flies rescued the loss of Δψm and decreased brain ROS levels (Fig. 8A,B). The expression of *S6kII* or *RSK4* also decreased the number of apoptotic cells in the brains of S6kII KO flies (Fig. 8C). Next, we analysed the effects of expression of either *S6kII* or *RSK4* on muscle degeneration in S6kII KO flies and found that the re-expression of either *S6kII* or *RSK4* decreased the number of TUNEL-positive apoptotic fibres (Fig. 8D). Finally, we tested the consequences of expressing either *S6kII* or *RSK4* on the lifespan of S6kII KO flies. We found that their re-expression prolonged the survival of S6kII KO flies (Fig. 8E,F). We conclude that the expression of S6kII or RSK4, a human orthologue, is sufficient to reverse the mitochondrial toxicity caused by the loss of this kinase in *Drosophila*.

## DISCUSSION

Mitochondria constantly fuse and divide to maintain their function and support overall cellular health. Interconnected mitochondria, which result from fusion, provide protection; in contrast, fission separates damaged parts, possibly causing apoptosis. Drp1 plays a crucial role in mitochondrial fission. Its activity leads to the fragmentation of mitochondria. ERK signalling can activate Drp1, promoting mitochondrial fission. This activation might occur through the direct phosphorylation of Drp1 (Kashatus et al., 2015) or via downstream effectors that regulate its activity. RSK family members interact with and regulate ERK signalling. As mentioned above, S6kII in *Drosophila* acts as a cytoplasmic anchor for ERK, modulating its activity. If this function is conserved in human RSKs, suppression of RSK1–3 might lead to dysregulated ERK signalling, which might indirectly affect mitochondrial dynamics by altering the phosphorylation status of Drp1. We did not observe significant alterations in the levels of Drp1 in the S6kII KO flies (Tables S1 and S2). We also attempted to measure the levels of Drp1 and its phosphorylated form in the lysate of *Drosophila* S6kII mutants using western blot analysis. However, we failed to detect Drp1 or its phosphorylated form by western blot analysis in flies. Thus, a role for RSKs in altering mitochondrial dynamics through the ERK‒Drp1 axis remains possible.

Disrupted mTORC1 (also known as Crtc in fly)-mediated mitochondrial protein translation could explain the altered proteome observed in S6kII knockout flies. A decrease in S6kII levels likely reduces mTORC1 signalling, subsequently downregulating essential nuclear-encoded mitochondrial proteins and resulting in mitochondrial fragmentation and decreased ATP production. We need to determine the activity of mTORC1 in flies lacking S6kII to understand this mechanism; one approach is to analyse the levels of 4E-BP (also known as Thor in fly) phosphorylation. If the status of 4E-PB was altered in S6kII KO flies, determining whether 4E-BP knockdown in these flies could rescue their mitochondrial defects would be interesting. We could also use polysome profiling to measure the translation status of mitochondrial mRNAs in S6kII KO flies and confirm that the decrease in mitochondrial protein levels observed in S6kII KO flies was linked to altered translation.

We observed the largest increase in brain ROS in adult *S6kII* mutants in a group of cells of the adult *Drosophila* brain (Fig. 3B). The precise nature of these cells remains uncharacterised. The identity of the specific types of cells with increased ROS levels following loss of S6kII requires further investigation.

We found that, in *S6kII* mutants, the mitochondrial area was decreased in neurons but increased in the IFM. The decrease in mitochondrial area in neurons is likely due to mitochondrial fragmentation. In neurons, mitochondria are actively transported and undergo fission/fusion in response to local energy demands. The loss of S6kII likely disrupts fission machinery (e.g. Drp1), causing fragmentation, whereas in muscle mitochondria this transport is absent. The IFM in *Drosophila* is a continuous cytoplasmic network formed by the fusion of myoblasts, unlike the compartmentalised mitochondria in neurons. Loss of S6kII likely causes IFM degeneration by compromising mitochondrial function. Defective mitochondria in *S6kII* mutants might disrupt the mechanical constraints that normally regulate mitochondrial size and positioning in the IFM (Katti et al., 2022). This leads to the mitochondrial swelling normally observed in the IFM in *Drosophila* with compromised mitochondrial function (Greene, 2003).

RSKs also regulate the activity of transcription factors, such as cAMP response element-binding protein (CREB). RSK isoforms, including RSK1 and RSK2, are activated downstream of ERK/MAPK signalling and directly phosphorylate CREB at Ser133. This phosphorylation regulates the transcriptional activity of CREB in response to growth factors and neuronal stimuli (reviewed in Lonze and Ginty, 2002). Phosphorylation of CREB by RSKs promotes the expression of genes involved in neuronal survival, plasticity and memory formation. Thus, RSKs serve as a critical link between extracellular signals and nuclear transcriptional responses, with direct relevance to brain function and neurodegeneration.

Loss of RSK function removes a key negative feedback mechanism on the ERK pathway, resulting in sustained or aberrant ERK activation (Nett et al., 2018). Elevated ERK activity has been shown to disrupt mitochondrial function. In neurons, excessive ERK signalling can lead to mitochondrial hyperpolarisation, increased production of ROS and impaired mitochondrial transport, contributing to synaptic dysfunction and neuronal death (Beck et al., 2015; He and Aizenman, 2010). In *Drosophila* and mammalian models, RSK deficiency causes redistribution of activated ERK from synaptic terminals to neuronal somata, correlating with defects in mitochondrial trafficking and synaptic transmission (Beck et al., 2015). Furthermore, chronic ERK activation can modulate mitochondrial permeability transition pore opening, ATP production and apoptosis, linking abnormal ERK signalling directly to mitochondrial and cellular dysfunction (He and Aizenman, 2010; Rasola et al., 2010). These findings indicate that RSK-mediated ERK feedback helps to maintain mitochondrial integrity and neuronal health.

Because the expression of *RSK4* rescued the mitochondrial defects observed in *S6kII* KO flies, we propose that *Drosophila* can be used as a model to study the consequences of polymorphisms in *RSK4* that are associated with human diseases. We did not test rescuing *S6kII* KO flies with other human orthologues. This analysis may be of interest because RSK isoforms have biological functions that are sometimes antagonistic (Chrysostomou et al., 2021). Our work establishes a pipeline to determine whether the expression of other orthologues – *RSK1*, *RSK2* and *RSK3* – can also rescue the phenotype of *S6kII* KO flies.

## MATERIALS AND METHODS

### Genetics and *Drosophila* strains

Fly stocks and crosses were maintained on standard cornmeal agar media at 25°C. The strains used were *elav*GAL4 (pan-neuronal driver) and *S6kII^KO^/FM7a* (Bloomington *Drosophila* Stock Center). HA-tagged cDNA fragments encoding full-length S6kII and RSK4 were subsequently cloned and inserted into the pUASTattB vector for PhiC31-mediated site-directed transgenesis. Transgenic flies were generated at the Cambridge Fly Facility, Department of Genetics, University of Cambridge (Cambridge, UK). All the experiments on adult flies were performed with males.

### Cell culture

U2OS cells (ATCC, HTB-96) were cultured in Dulbecco's modified Eagle medium (Gibco, 11965092) supplemented with 10% foetal bovine serum and 1% penicillin‒streptomycin at 37°C in a humidified incubator with 5% CO₂. The cells were passaged every 2–3 days upon reaching 80–90% confluence using 0.25% trypsin–EDTA. For the experiments, the cells were seeded at an appropriate density and allowed to adhere overnight before treatment or transfection. The cells tested negative for mycoplasma, and their identity was validated by short tandem repeat genotyping.

### Transfections

The cells were seeded in a 24-well plate at a density of 50,000 cells per well and allowed to adhere overnight. The next day, the cells were transfected with different siRNAs (*RSK1* siRNA, Horizon Discovery, M-003025-04-0005; *RSK2* siRNA, Horizon Discovery, M-003026-02-0005; *RSK3* siRNA, Horizon Discovery, M-004663-02-0005; *RSK4* siRNA, Horizon Discovery, M-004670-01-0010; and negative control siRNA, Qiagen, 1027281) using Lipofectamine RNAiMax (Invitrogen, 13778100) according to the manufacturer's instructions. After 24 h of incubation, the medium was replaced with fresh complete culture medium, and the cells were harvested after 3–4 days for RNA extraction and confocal imaging analysis.

### Lifespan analysis

Groups of 12 newly eclosed males of each genotype were placed into separate vials with food and maintained at 25°C. The flies were transferred to vials containing fresh food every 2–3 days, and the number of dead flies was recorded. The data are presented as Kaplan‒Meier survival distributions, and significance was determined with log-rank tests.

### Climbing assay

Motor performance was assessed as previously described (Hardy et al., 2023). Briefly, 20 male flies were placed into glass columns (23 cm long, 2.5 cm in diameter) lined with nylon mesh (250 µm, Dutscher Scientific) and marked with a line at 15 cm. After a 30–60 min recovery from CO_2_ anaesthesia, the flies were gently tapped to the bottom of the vials, and the time required for five flies to climb above the marked line was recorded. For each experiment, at least three cohorts of 20 flies from each genotype were scored (with three individual biological replicates for each group). The climbing latency was defined as the average time in seconds for five flies to climb above the marked line, with averages being pooled together from each group of 20 flies per genotype.

### Antibodies and dyes

The primary antibodies used in this study included anti-DJ-1 (1:1000, Imgenex, IMG-3038), anti-Ref(2)P (1:1000, Abcam, ab178440), anti-Atg8 (1:1000, Sigma-Aldrich, ZRB1585), mono- and poly-ubiquitinylated conjugate monoclonal antibodies (FK2, 1:1000, Enzo Life Sciences, BML-PW8810), anti-ATP5A (1:100, Abcam, ab14748) and anti-α-Tubulin (1:1000, Sigma-Aldrich, T6074). The dyes used were phalloidin (1:500, Thermo Fisher Scientific, A12379) and Hoechst 33342 (1:2000, Thermo Fisher Scientific, 33342). The secondary antibodies used were Alexa Fluor™ 568-conjugated goat anti-mouse (1:250, Invitrogen, A11031), Alexa Fluor™ 488-conjugated goat anti-mouse (1:250, Invitrogen, A11029), and 800CW-conjugated donkey anti-mouse (1:1000, Licor, 926-32213) and anti-rabbit (1:1000, Licor, 926-32213) antibodies.

### Protein extraction and immunoblotting

Protein extracts from whole flies or dissected heads were prepared by grinding the flies in lysis buffer (150 mM NaCl, 1% Triton X-100, 0.5% sodium deoxycholate, 0.1% SDS, 50 mM Tris-HCl, pH 7.5) with protease inhibitors (Thermo Fisher Scientific, 11834101) and phosphatase inhibitors (Roche, 4906845001). The suspension was collected by centrifugation at 14,000 ***g*** for 10 min at 4°C. Protein concentration was determined using bicinchoninic acid (BCA) assay (Thermo Fisher Scientific, 23225). Equal amounts of protein were mixed with 4× LDS loading buffer (Bio-Rad, 1610747), followed by denaturation at 95°C for 5 min.

For immunoblotting, the samples were loaded onto an SDS‒PAGE gel for electrophoresis. After separation, the proteins were transferred onto a PVDF membrane via a wet transfer system, followed by blocking with 5% nonfat milk in TBST (Tris-buffered saline with Tween 20) for 1 h at room temperature. The membranes were subsequently incubated with the indicated primary antibody at 4°C overnight before being incubated with the secondary antibody for 2 h at room temperature. Signals were visualised using a Licor imaging system.

### Immunohistochemistry and TUNEL

For imaging, thoraxes and brains were dissected from live flies in PBS, fixed with 4% paraformaldehyde for 30 min and blocked overnight in blocking buffer (10% normal goat serum in PBS/0.5% Triton X-100). The samples were then incubated with primary antibodies [anti-ATP5A (1:100, Abcam, ab118482) and anti-Ref(2)P (1:100, Abcam, ab178440)] at 4°C overnight, followed by incubation with secondary antibodies [Alexa Fluor™ 568-conjugated goat anti-mouse antibody (1:250, Invitrogen, A11031), Alexa Fluor™ 488-conjugated goat anti-mouse antibody (1:250, Invitrogen, A11029)] and phalloidin (1:500, Thermo Fisher Scientific, A12379) for 2 h at room temperature. Confocal images were acquired with a Zeiss LSM 880 confocal microscope. TUNEL staining was performed with an ApopTag red *in situ* apoptosis detection kit (Merck, S7165) according to the manufacturer's instructions. The quantitative assessment of cell death in brains was performed in single anatomically matched coronal sections of individual brains. Sections were selected at a consistent depth to include central neuropil regions. We determined the ratio of TUNEL- to Hoechst-positive cells, as previously described (Elguero et al., 2023). The quantitative assessment of cell death in muscle fibres was performed by calculating the ratio of TUNEL-positive cells to the total number of muscle fibre-positive region, as previously described (Yang et al., 2022).

### Microscopy-based assessment of mitochondrial function and morphology

The Δψm in *Drosophila* adult brains was measured as previously described (Tufi et al., 2014). Briefly, adult brains were loaded with 40 nM TMRM in loading buffer (10 mM HEPES, pH 7.35, 156 mM NaCl, 3 mM KCl, 2 mM MgSO_4_, 1.25 mM KH_2_PO_4_, 2 mM CaCl_2_ and 10 mM glucose) for 40 min at room temperature, and the dye was present during the experiment. In these experiments, TMRM was used in redistribution mode to assess the Δψm. Therefore, a reduction in TMRM fluorescence represents mitochondrial depolarisation. Confocal images were obtained using a Zeiss LSM 880 confocal microscope equipped with a 20× air objective. The illumination intensity was maintained at a minimum (0.1–0.2% of the laser output) to avoid phototoxicity, and the pinhole was set to acquire an optical slice of 2 μm. The fluorescence was quantified by exciting TMRM with a 565 nm laser and measuring it above 580 nm. *Z*-stacks of five 300-μm^2^ fields per brain were acquired, and the mean maximal fluorescence intensity was measured for each group.

The adult brains were dissected in cold PBS and incubated with 5 μM MitoSOX Red mitochondrial superoxide indicator (Molecular Probes, M36008) for 20 min to measure ROS levels. After the incubation, the samples were washed with PBS for 10 min and immediately imaged with a Zeiss LSM880 confocal microscope. The maximal-intensity projections of the MitoSOX Red signal in the fly brain were quantified using ImageJ.

The mitochondrial length was quantified in the mechanosensory axons of the ventral nerve cord (VNC) from third-instar larvae. The VNC was dissected in PBS, transferred to a drop of PBS as a mounting medium on glass slides, covered with a coverslip and captured with a Zeiss LSM880 confocal microscope. The mitochondrial length was calculated using the ‘segmented line’ tool in ImageJ to measure the length of mitoGFP-positive mitochondria across their largest dimension. *Z*-projection of 10-µm-thick stacks was used to monitor the 3D distribution of mitochondria and measure lengths more accurately.

### Transmission electron microscopy

For transmission electron microscopy, adult thoraces were fixed for 2 h with 0.1 M sodium cacodylate buffer (pH 7.4) containing 2% paraformaldehyde, 2.5% glutaraldehyde and 0.1% Tween 20. The samples were subsequently fixed for 1 h at room temperature in a solution containing 1% osmium tetroxide and 1% potassium ferrocyanide. After fixation, the samples were stained en bloc with 5% aqueous uranyl acetate overnight at room temperature; then, they were dehydrated by washing with a series of ethanol solutions and embedded in TAAB epoxy resin (TAAB Laboratories Equipment, Aldermaston, UK). Semithin sections were stained with Toluidine Blue, and areas of the sections were selected for ultramicrotomy. Ultrathin sections were stained with lead citrate and imaged using a TemCam XF416 digital camera and EM Menu software (TVIPS, Gilching, Germany) with a Joel 1400 electron microscope (Jeol UK, Welwyn Garden City, UK).

### Citrate synthase assay

Citrate synthase activity was measured using a Citrate Synthase Assay Kit (Sigma-Aldrich, CS0720). Mitochondria were extracted from 20 male flies (5 days old or 30 days old) using a mitochondria isolation kit (Sigma-Aldrich, MITOISO2-1KT) according to the manufacturer's instructions. The protein concentrations were determined by BCA assay (Thermo Fisher Scientific, 23225). The sample was resuspended in CelLytic M and supplemented with assay buffer, 30 mM acetyl-CoA solution and 10 mM 5,5′-dithiobis-(2-nitrobenzoic acid) solution. The baseline reaction was measured at 412 nm for 1.5 min using an M200PRO plate reader (TECAN, Männedorf, Switzerland). Following the addition of 10 mM oxaloacetic acid solution, the absorbance of the mixture was measured for 1.5 min. The absorbance values were plotted over time (min) for each reaction, and the change in absorbance (ΔA412/min) was used to determine citrate synthase activity with the following equation:

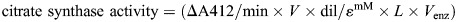
where *V*=the reaction volume (ml), dil=the dilution factor of the original sample, ε^mM^=13.6 mM^−1^ cm^−1^, *L*=the length of the cuvette (cm), and *V*_enz_=the volume of the sample (ml). Units of citrate synthase activity (μmol/ml/min) were normalised to the protein concentration (mg/ml).

### Proteomic profiling

A proteomic analysis was performed on whole flies. Briefly, each biological replicate consisted of 20 adult male flies from either the 5-day-old or 35-day-old groups, which were processed separately. We processed five biological replicates for each sample. Fly samples were homogenised in RIPA buffer (150 mM NaCl, 1% Triton X-100, 0.5% DOC, 0.1% SDS, 50 mM Tris-HCl, pH 7.5) supplemented with protease inhibitors (Thermo Fisher Scientific, 11834101) and phosphatase inhibitors (Roche, 4906845001) on ice using a probe sonicator (3 s on/6 s off, 2 min). The lysates were incubated on ice for 30 min with intermittent vortexing and then centrifuged at 12,000 ***g*** for 15 min at 4°C to collect the supernatant. Protein concentration was determined using a BCA protein assay kit (Thermo Fisher Scientific, 23225). Protein samples were reduced with 10 mM dithiothreitol at 37°C for 1 h, alkylated with 20 mM iodoacetamide in the dark for 30 min at room temperature, and digested overnight with trypsin (1:50, w/w) at 37°C. The digestion was stopped by adding 0.1% formic acid.

TMTpro 32 plex reagents (Thermo Fisher Scientific, A40000839) were equilibrated to room temperature and reconstituted in anhydrous acetonitrile. Each peptide sample was labelled with a distinct TMTpro tag by incubation at room temperature for 1 h, and the reaction was quenched with 5% hydroxylamine for 15 min. Labelled samples were pooled, dried using a vacuum concentrator and desalted with C18 solid-phase extraction columns. The pooled TMT-labelled peptides were fractionated using high-pH reverse-phase chromatography on a C18 column. Peptides were separated into eight to 12 fractions using an increasing gradient of acetonitrile in 10 mM ammonium formate (pH 10.0) and dried under vacuum.

The fractions were analysed using a nano-LC system coupled to a Thermo Fisher Scientific Orbitrap mass spectrometer. The samples were injected into an Ultimate 3000 RSLC™ nanosystem (Thermo Fisher Scientific) coupled to an analytical Orbitrap Eclipse™ mass spectrometer (Thermo Fisher Scientific). The ion data were analysed using three high-field asymmetric waveform ion mobility spectrometry (FAIMS) compensation voltages (−45 V, −60 V and −75 V), and each FAIMS experiment had a maximum cycle time of 2 s. For each FAIMS experiment, the data-dependent SPSMS3 RTS program used for data acquisition consisted of a 120,000-resolution full-scan mass spectrometry scan [automatic gain control set to 50% (200,000 ions) with a maximum fill time of 30 ms] using a mass range of 415–1500 m/z. The raw data were imported and analysed with Proteome Discoverer v2.5 (Thermo Fisher Scientific). The raw files were subjected to a database search using Proteome Discoverer with Sequest HT against the *Drosophila* reference proteome (UniProt UP000000803, containing 21,135 entries, accessed on 4 August 2022). We processed the peptide spectral match (PSM) level data using the standard camprotR pipeline. Quality control and filtering were performed on the PSMs, and normalisation was performed at the protein level.

### Statistical analysis

Statistical analyses were performed using GraphPad Prism. The data are presented as the mean values, and the error bars indicate s.e.m. or s.d. In the violin plots, the solid line represents the median, whereas the dashed lines represent the quartiles. The number of biological replicates per experimental variable (*n*) is indicated in the respective figure or figure legend. No sample was excluded from the analysis, unless stated otherwise. Masking was not performed. For all the statistical analyses, the D'Agostino and Pearson tests were used to determine whether the data followed a normal distribution. Based on the normality test results, a parametric or nonparametric analysis was used. *P*≤0.05 was considered significant.

### Digital image processing

The fluorescence images were acquired as uncompressed bitmapped digital data (ZVI or TIFF format) and processed using Fiji with established scientific imaging workflows. Confocal images acquired with identical settings were processed via a five-tone heatmap in Fiji to visualise the pixel intensity.

## Supplementary Material

10.1242/dmm.052374_sup1 Supplementary information

Table S1. Full complement of changes in protein levels in 5-day-old S6kII KO adult flies. The individual levels of proteins (logFoldChange_d5) are quantified as log base 2 intensity values from the mass spectrometer. padjBH_d5, *P* value corrected using the Benjamini-Hochberg method. This table is related to Fig. 6.

Table S2. Full complement of changes in protein levels in 35-day-old S6kII KO adult flies. The individual levels of proteins (logFoldChange_d35) are quantified as log base 2 intensity values from the mass spectrometer. padjBH_d35, *P* value corrected using the Benjamini-Hochberg method. This table is related to Fig. 6.

Table S3. List of altered proteins in 5-day-old S6kII KO adult flies mapped to the mitochondrial electron transport Gene Ontology term (GO:0006123). The individual levels of proteins (logFoldChange_d5) are quantified as log base 2 intensity values from the mass spectrometer. padjBH_d5, *P* value corrected using the Benjamini-Hochberg method. This table is related to Fig. 6.

Table S4. List of altered proteins in 35-day-old S6kII KO adult flies mapped to the respiratory electron transport chain Gene Ontology term (GO:0022904). The individual levels of proteins (logFoldChange_d35) are quantified as log base 2 intensity values from the mass spectrometer. padjBH_d35, *P* value corrected using the Benjamini-Hochberg method. This table is related to Fig. 6.

Table S5. List of the 49 filtered proteins in 5-day-old S6kII KO flies used for the network analysis. The individual levels of proteins (logFC_d5, filtered at a threshold of 2-fold) are quantified as log base 2 intensity values from the mass spectrometer and filtered for significance (padjBH ≤ 0.05). This table is related to Fig. 6.

Table S6. List of the 76 filtered proteins in 35-day-old S6kII KO flies used for the network analysis. The individual levels of proteins (logFC_d35, filtered at a threshold of 2-fold) are quantified as log base 2 intensity values from the mass spectrometer and filtered for significance (padjBH ≤ 0.05). This table is related to Fig. 6.
