# Supplementary material for: Loss of Drosophila ribosomal protein S6 kinase II causes mitochondrial dysfunction and cell death
Source: Dis Model Mech. 2025 Aug 19;18(8):dmm052374. doi: 10.1242/dmm.052374 (PMC12403519; doi:10.1242/dmm.052374)
Supplement: Supplementary information [file dmm-18-052374-s1.pdf]

Table S1. Full complement of changes in protein levels in 5-day-old S6kII KO adult flies.

The individual levels of proteins (logFoldChange_d5) are quantified as log base 2 intensity values from the mass spectrometer. padjBH_d5, *P* value corrected using the Benjamini–Hochberg method. This table is related to Fig. 6.

Available for download at
https://journals.biologists.com/dmm/article-lookup/doi/10.1242/dmm.052374#supplementary-data

Table S2. Full complement of changes in protein levels in 35-day-old S6kII KO adult flies.

The individual levels of proteins (logFoldChange_d35) are quantified as log base 2 intensity values from the mass spectrometer. padjBH_d35, *P* value corrected using the Benjamini–Hochberg method. This table is related to Fig. 6.

Available for download at
https://journals.biologists.com/dmm/article-lookup/doi/10.1242/dmm.052374#supplementary-data

Table S3. List of altered proteins in 5-day-old S6kII KO adult flies mapped to the mitochondrial electron transport Gene Ontology term (GO:0006123). The individual levels of proteins (logFoldChange_d5) are quantified as log base 2 intensity values from the mass spectrometer. padjBH_d5, *P* value corrected using the Benjamini–Hochberg method. This table is related to Fig. 6.

Available for download at
https://journals.biologists.com/dmm/article-lookup/doi/10.1242/dmm.052374#supplementary-data

Disease Models & Mechanisms • Supplementary information

Table S4. List of altered proteins in 35-day-old S6kII KO adult flies mapped to the respiratory electron transport chain Gene Ontology term (GO:0022904). The individual levels of proteins (logFoldChange_d35) are quantified as log base 2 intensity values from the mass spectrometer. padjBH_d35, *P* value corrected using the Benjamini–Hochberg method. This table is related to Fig. 6.

Available for download at
https://journals.biologists.com/dmm/article-lookup/doi/10.1242/dmm.052374#supplementary-data

Table S5. List of the 49 filtered proteins in 5-day-old S6kII KO flies used for the network analysis.
The individual levels of proteins (logFC_d5, filtered at a threshold of 2-fold) are quantified as log base 2 intensity values from the mass spectrometer and filtered for significance (padjBH $\leq$ 0.05). This table is related to Fig. 6.

Available for download at
https://journals.biologists.com/dmm/article-lookup/doi/10.1242/dmm.052374#supplementary-data

Table S6. List of the 76 filtered proteins in 35-day-old S6kII KO flies used for the network analysis.
The individual levels of proteins (logFC_d35, filtered at a threshold of 2-fold) are quantified as log base 2 intensity values from the mass spectrometer and filtered for significance (padjBH $\leq$ 0.05). This table is related to Fig. 6.

Available for download at
https://journals.biologists.com/dmm/article-lookup/doi/10.1242/dmm.052374#supplementary-data
